# Living With the Dead: A Case Report of Chronic Schizophrenia Associated With Prolonged Cohabitation With a Decomposed Corpse in Morocco

**DOI:** 10.7759/cureus.115204

**Published:** 2026-08-26

**Authors:** Fouad Laboudi, Zineb Bencharfa, Soukaina Bellafqih, Abderrazzak Ouanass

**Affiliations:** 1 Psychiatry and Behavioral Sciences, Faculty of Medicine and Pharmacy/Mohammed V University of Rabat, Rabat, MAR; 2 Psychiatry, Faculty of Medicine and Pharmacy/Mohammed V University of Rabat, Rabat, MAR; 3 Psychiatry, Ar-Razi University Psychiatric Hospital in Salé, Salé, MAR; 4 Psychiatry, Ar-Razi University Psychiatric Hospital in Salé, Ibn Sina University Hospital Center, Salé, MAR

**Keywords:** cohabitation with the dead, delayed body discovery, forensic psychiatry, psychosis, schizophrenia, social isolation

## Abstract

The discovery of decomposed human remains in private residences is a relatively common occurrence in forensic practice. However, finding a living individual cohabiting with a corpse represents an exceptionally rare medico-legal situation, raising complex psychiatric, forensic, and ethical issues. Such cases are most often associated with severe mental illness, social isolation, or pathological grief.

We report the case of a 55-year-old unmarried former military serviceman with a long-standing history of schizophrenia who was brought to the psychiatric emergency department under police requisition after being found living with the decomposing body of his brother. Following a report of a foul odor, authorities entered the home and discovered the patient’s brother’s decomposed body after the patient threatened them with a knife and exhibited bizarre behavior. Upon examination, the patient exhibited severe psychotic symptoms, including persecutory delusions, auditory hallucinations, thought disorganization, impaired insight, and marked behavioral disturbances. The deceased brother had apparently remained undiscovered for 15 days, while the patient continued to occupy the residence without notifying authorities. A multidisciplinary forensic and psychiatric assessment was conducted to clarify the circumstances surrounding the delayed discovery and to evaluate the patient’s mental state.

Cohabitation with a deceased individual should prompt systematic psychiatric evaluation whenever severe mental illness is suspected. The early identification of vulnerable individuals living in social isolation may help prevent delayed corpse discovery and improve both public health and forensic outcomes.

## Introduction

The discovery of bodies in an advanced state of decomposition in a residence is a relatively common occurrence in forensic medicine [1]. These cases complicate identification, determining the cause of death, and estimating the postmortem interval and pose investigative challenges due to a lack of evidence, requiring a multidisciplinary approach in forensic science [2]. Estimating the postmortem interval is difficult in cases of delayed discovery of death. Several methods are used for this purpose [3]. Postmortem biological processes, which are influenced by various factors, are crucial for medical examiners [4]. It is vital to distinguish between postmortem changes and vital lesions in order to make reliable assessments [5]. Social isolation, which is common in modern urban society, particularly affects people with mental illness, increasing their vulnerability and mortality [6].

The discovery of a body at home is usually linked to isolation or relational withdrawal, but a relative may continue living with the body for days to months because of denial, pathological grief, or psychiatric illness; reported associations include shared psychosis, personality disorders, extreme family isolation, and complicated grief.

Prolonged cohabitation with a corpse (“living with the dead”) is a rare phenomenon that has been little described in the forensic literature. It is not uncommon to find bodies in urban apartments, a phenomenon closely linked to social isolation among people with mental illness, a feature increasingly common in contemporary urban society and associated with elevated mortality, leaving this population particularly vulnerable to undetected death. Such cases can be extremely difficult to investigate given the lack of available evidence, requiring a multidisciplinary approach [7].

People with mental illness who live alone face difficulties related to their whereabouts and access to help during a crisis; they often die without their deaths being discovered promptly. Several factors contribute to this isolation, notably limited communication with family and the community [6,8,9]. Indeed, social isolation is associated with a significant increase in mortality [10]. The lack of visits has been identified as a key factor contributing to delays in discovering deaths, suggesting that community-based strategies, such as welfare checks by neighbors, could reduce these delays [11]. The data show a link between a prolonged delay in discovering deaths and reduced communication with family members [12].

Psychotic disorders are now defined, with major symptoms specified as persecutory delusions, auditory hallucinations, disorganized thinking, impaired insight, and marked behavioral disturbance [13]. It can profoundly impair judgment, awareness of reality, and reactions to the death of a loved one. Individuals with psychotic disorders face numerous obstacles. It is essential to adopt a holistic approach to services to ensure that their basic daily needs and social engagement are met, without neglecting their significant mental and physical health needs [14]. The assessment requires collaboration among forensic pathologists, psychiatrists, anthropologists, entomologists, biologists, and investigators.

A case is presented in which a man lived with his brother’s body, illustrating the phenomenon of “living with a dead person.” Previous reports have described similar situations, including Cortellini et al. [6], who reported an elderly woman living with her husband’s mummified/skeletonized remains, and Bosco et al. [8], who described two cases of prolonged cohabitation with mummified remains.

Our case differs from these reports by involving chronic schizophrenia and raises several important medico-legal issues, including mental capacity, legal responsibility, the circumstances of death, access to psychiatric care, and the protection of vulnerable individuals. The challenges associated with social isolation and its impact on forensic science and contemporary society are also examined.

## Case presentation

The patient, a 55-year-old single man, was admitted to the psychiatric ward at the request of the police due to death threats, aggressive behavior, and incoherent remarks.

According to the police report, neighbors alerted the authorities after noticing a strong, foul odor coming from the patient’s home. The police attempted to enter the house, but the patient refused to open the door and threatened them with a knife. After forcing their way in, the police discovered the body of the patient’s brother, in an advanced state of decomposition, inside the house, with no evidence of concealment or scene alteration; the body was found in the position/environment in which it was located at discovery. The patient had been living alone with the corpse for approximately 15 days and was subsequently transferred for a psychiatric evaluation.

According to information gathered from his relatives, he has a history of schizophrenia since 1998; no other chronic/major medical condition, hospitalization, surgery, or trauma documented; no reported substance use; prior antipsychotic regimen; and no available records of psychiatric/social-service follow-up details. The patient came from a family in a situation of social vulnerability. He left school after elementary school; after that, he subsequently joined the military but was discharged from the army, after which he worked as a mason’s assistant at a construction company. Psychotic symptoms reportedly began 28 years ago, when he developed strange and disorganized behavior, along with incoherent and delusional remarks directed at his coworkers, which led to his dismissal on medical grounds. He was hospitalized once in 1998 and treated with antipsychotics, resulting in partial improvement (agitation and behavioral disturbance decreased, but delusional ideas and thought disorder persisted partially).

In the years that followed, psychiatric follow-up was irregular, with poor treatment adherence and multiple psychotic relapses characterized by aimless wandering, social withdrawal, delusions of persecution, self-neglect, monologues, and inappropriate laughter. At the age of 47, he lost his father, and due to this event, treatment was completely discontinued, and his clinical condition gradually deteriorated. At 50 years old, his mother died, and the patient continued to live with his brother, who was also reportedly diagnosed with schizophrenia. He and his deceased brother, aged 52 years, shared a close cohabiting relationship; both brothers received regular financial support from a cousin; the emotional nature and the evolution of this relationship over time were not sufficiently documented.

Upon admission, the patient appeared emaciated and visibly neglected: dirty clothes, poor hygiene, long and unkempt hair and beard, dirty fingernails, and a strong body odor. Although calm and cooperative, he made only superficial contact. Physical and neurological examination was performed; no focal neurological signs or significant somatic abnormality was found. He was fully oriented in time and space, despite being easily distracted. Vital signs were stable on admission, with no significant abnormality. His speech was highly disorganized, with digressions, interruptions, blocks, and off-topic responses. Exploring his thoughts was difficult due to a marked formal thought disorder. He provided limited information, of reduced reliability given his psychiatric state. Although he denied experiencing any hallucinations, he frequently appeared to react to internal stimuli, adopting listening postures and engaging in monologues. His affect was blunted, and his judgment and insight into his illness were profoundly impaired.

The overall clinical picture was consistent with schizophrenia, a diagnosis based on the DSM-5-TR criteria, characterized by severe psychosocial deterioration, poor treatment adherence, and a significant impairment of the sense of reality [13]. The Positive and Negative Syndrome Scale (PANSS) total score was 105, with predominant positive symptoms and general psychopathology, consistent with substantial psychotic symptomatology. He had comprehensive laboratory results; all were normal or negative (CRP, white blood cell {WBC}, neutrophils, hemoglobin, blood/urine cultures, HIV, hepatitis B surface antigen {HBsAg}, anti-hepatitis C virus {HCV}, and *Treponema pallidum* hemagglutination assay {TPHA}/venereal disease research laboratory {VDRL}) (Table 1). Radiological workup was also performed, with no imaging abnormality that could explain or contribute to the psychiatric symptoms.

**Table 1:** Laboratory results HBsAg, hepatitis B surface antigen; HCV, hepatitis C virus; Ab, antibody; TPHA, *Treponema pallidum* hemagglutination assay; VDRL, venereal disease research laboratory; ECBU, Cytobacteriological Examination of Urine

Test	Result	Unit	Reference range
CRP	3.2	mg/L	<5
White blood cell count	7.8	G/L	4.0-10.0
Neutrophils	4.6	G/L	1.5-7.5
Hemoglobin	14.2	g/dL	13.0-17.0
Blood cultures	Negative	-	Negative
Urine culture (ECBU)	Negative	-	Negative
HIV (Ag/Ab)	Negative	-	Negative
HBsAg	Negative	-	Negative
Anti-HCV antibodies	Negative	-	Negative
TPHA/VDRL	Negative	-	Negative

The patient received multidisciplinary psychiatric care including regular interviews, daily clinical monitoring, reassurance, psychoeducation, and psychosocial support throughout admission, alongside pharmacotherapy: olanzapine 20 mg/day (second-generation atypical antipsychotic, D2/5-HT2A antagonist) plus levomepromazine 200 mg/day (first-generation phenothiazine, D2 antagonist) adjunctive for sedation; he was monitored clinically for sedation, orthostatic hypotension, anticholinergic effects, and extrapyramidal symptoms (EPS) during titration; no significant EPS were observed, and he remained hospitalized for 27 days with significant improvement: reduction in agitation, aggressiveness, and behavioral disturbance, with better cooperation and less disorganized speech; hallucinations and delusions decreased but did not fully resolve, and residual delusional ideas and partial insight impairment persisted.

He was discharged to a social services center for outpatient follow-up, the assessment of social/family situation, help accessing available resources, and referral to appropriate follow-up structures, to support the continuity of care and reduce isolation. Outpatient follow-up was every two weeks initially and then monthly, for approximately six months. At the last visit, he had stabilized, with reduced agitation but persistent residual delusions and partial insight; quality of life was stable but limited by isolation and reduced autonomy.

## Discussion

The prolonged cohabitation of a living individual with the corpse of a deceased loved one (“living with the dead”) represents an atypical case in forensic medicine. Although the delayed discovery of death is common in forensic medicine, situations in which a person lives with a deceased body remain rare and are generally described only through individual case reports. In our modern, urban society, this is often an indicator of social isolation. This rarity explains the lack of specific recommendations and underscores the scientific significance of our observation [8].

Our patient exhibited several factors that recur in the few published cases: chronic schizophrenia that had been progressing for more than 28 years, a prolonged interruption in psychiatric care, marked anosognosia, significant social isolation, and socioeconomic vulnerability [6,8]. These factors are recognized as important determinants of psychosocial disorganization and the progressive loss of adaptive abilities in patients with schizophrenia [15,16]. The impaired judgment, formal thought disorders, and loss of contact with reality observed in our patient likely constitute the primary mechanisms underlying his inability to acknowledge his brother’s death or to engage in socially appropriate behavior. Bosco et al. have described similar situations in which psychotic patients continued to interact with the deceased due to psychotic denial or profound cognitive disorganization [8]. In such a context, the difficulties associated with the illness may accumulate within the same household, including impaired social and family functioning, social withdrawal, difficulties in carrying out daily activities, the limited use of healthcare services, and mutual dependence. The absence of a relative able to recognize a deterioration in mental state or facilitate access to care may contribute to delays in detecting a crisis or a situation of neglect. In our case, the cohabitation of two brothers with schizophrenia may have constituted an additional vulnerability factor and may have contributed to the delayed discovery of the death. However, this hypothesis should be interpreted with caution, as the available data do not allow a direct causal link to be established between the psychiatric condition of the two brothers and the circumstances of the death or the delay in its discovery.

Unlike cases of the deliberate concealment of a corpse in a criminal context, our patient made no attempt to hide the body or alter the scene. This absence of organized behavior, combined with severe psychotic symptoms, points more toward a psychopathological incapacity than toward an intent to conceal. This distinction is of particular importance in forensic medicine, as it determines the legal interpretation of the facts, as well as the assessment of mental capacity.

Our observation also fits within the broader context of solitary deaths (*kodokushi*), a phenomenon initially described in Japan but now reported in many countries. Several recent studies show a steady increase in deaths discovered late, linked to an aging population, urbanization, and a decline in social interactions. Byard reported an increase in solitary deaths in Australia, rising from 1.6% of autopsies between 2000 and 2004 to nearly 10% between 2019 and 2023, illustrating the growing significance of this public health issue [17].

The factors associated with delays in discovering deaths are now well established: male gender, single status, social isolation, poverty, chronic illnesses, and severe psychiatric disorders [18]. Our patient exhibited most of these factors. However, unlike in classic cases of *kodokushi*, where the deceased lives alone, in this case, the deceased shared his home with his brother, who has schizophrenia. The delay in discovery was therefore not solely due to social isolation but also to the cohabitant’s inability to understand the situation and seek help. This particular circumstance places our case at the intersection of the “living with the dead” phenomenon and that of *kodokushi*.

The highly advanced state of decomposition of the body also posed a major challenge for the forensic investigation. Postmortem changes significantly limit the ability to identify the cause of death and reduce the accuracy of estimating the time of death. In such situations, current guidelines emphasize the need for a multidisciplinary approach combining autopsy, toxicology, anthropology, forensic entomology, and criminal investigation to improve the reconstruction of the circumstances surrounding the death [19,20].

Beyond the medico-legal aspects, this case highlights the consequences of a disruption in psychiatric care for people with chronic schizophrenia. The management of schizophrenia combines antipsychotic treatment, psychosocial interventions, and long-term follow-up. In vulnerable patients with limited autonomy, close coordination between psychiatric, family, and social services is essential to ensure the continuity of care and prevent crises or neglect.

Despite having been diagnosed long ago, our patient no longer received any specialized follow-up care after the death of his parents. This situation illustrates the limitations of community-based care systems in resource-constrained settings: outpatient follow-up, psychoeducation, psychosocial rehabilitation, assertive/mobile community treatment for high-vulnerability cases, and coordination with social services applied to this household’s context. International guidelines, however, emphasize the importance of mobile teams, home visits, and coordination among psychiatrists, primary care physicians, and social workers to prevent gaps in care, social isolation, and their consequences [16].

Finally, our observation serves as a reminder that the phenomenon of “living with the dead” cannot be interpreted solely from the perspective of individual psychopathology. It likely results from a complex interaction between severe mental illness, social vulnerability, isolation, precarious living conditions, and inadequate community support. This biopsychosocial approach provides a better understanding of how such exceptional situations arise and underscores the need for multicenter studies examining associated factors, including psychiatric illness, social isolation, family dependence, autonomy, and housing conditions, with particular attention to multi-affected households. It also highlights the importance of evaluating community-based care interventions and strengthening collaboration between psychiatry, forensic medicine, public health, and social services to prevent such situations.

## Conclusions

This case illustrates a particularly rare form of cohabitation with a corpse in a patient suffering from chronic schizophrenia. Its significance extends beyond the exceptional nature of the observation by highlighting the interactions between psychotic disorders, social isolation, and delayed discovery of death. It underscores the importance of a rigorous forensic psychiatric evaluation, a multidisciplinary approach to investigations, and the development of community psychiatry programs targeting the most vulnerable patients in order to prevent such situations. Finally, the scarcity of cases reported in the literature underscores the need for further research into the psychopathological mechanisms, social determinants, and medico-legal implications of the “living with the dead” phenomenon. A better understanding of this condition will help improve forensic evaluation, the care of patients with severe psychotic disorders, and the development of prevention strategies based on community psychiatry and the maintenance of social ties.
